# Observation of neutrophil extracellular traps in the development of diabetic nephropathy using diabetic murine models

**DOI:** 10.1186/s42826-024-00226-2

**Published:** 2024-11-07

**Authors:** You Hyun Jeon, Se-Hyun Oh, Soo-Jung Jung, Eun-Joo Oh, Jeong-Hoon Lim, Hee-Yeon Jung, Ji-Young Choi, Sun-Hee Park, Chan-Duck Kim, Yong-Lim Kim, Chang-Won Hong, Jang-Hee Cho

**Affiliations:** 1 Division of Nephrology, Department of Internal Medicine, Kyungpook National University Hospital, 130 Dongdeok-ro, Jung-gu, Daegu, 41944 South Korea; 2 Bio-Medical Research Institute, Kyungpook National University, Daegu, Republic of Korea; 3 Cell and Matrix Research Institute, Kyungpook National University, Daegu, Republic of Korea; 4 Department of Physiology, School of Medicine, Kyungpook National University, 680 Gukchaebosang-ro, Jung-gu, Daegu, 41944 Republic of Korea

**Keywords:** Chronic kidney disease, Diabetic nephropathy, Hyperglycemia, Neutrophil, Neutrophil extracellular traps

## Abstract

**Background:**

Diabetic nephropathy (DN) is a progressive complication among patients with diabetes and the most common cause of end-stage kidney disease. Neutrophil extracellular traps (NETs) are known to play a role in kidney disease, thus this study aimed to determine their role in the development of diabetic kidney disease using diabetic murine models.

**Results:**

Protein and histological analyses revealed that db/db mice and streptozotocin DN models expressed no significant NET-related proteins, myeloperoxidase, citrullinated histone H3 (citH3), neutrophil elastase, and lymphocyte antigen 6 complex locus G6D (Ly6G). However, the inflamed individuals in the DN model showed that citH3 and Ly6G were highly deposited in the renal system based on immunohistochemistry images. In vitro, NET treatment did not induce apoptosis in glomerular endothelial and renal tubular epithelial cells. NET inhibition by DNase administration demonstrated no significant changes in cell apoptosis.

**Conclusions:**

NET-related proteins were only expressed in the DN model with tubulointerstitial inflammation. Our study revealed that NETs are only induced in mice with hyperglycemia-induced inflammation.

**Supplementary Information:**

The online version contains supplementary material available at 10.1186/s42826-024-00226-2.

## Background

Diabetic nephropathy (DN) is a critical complication of diabetes mellitus and the leading cause of chronic kidney disease (CKD) [1, 2]. Clinical presentation of DN is characterized by proteinuria, and deterioration of kidney function. Despite early diagnosis and the development of new treatments, the prevalence of diabetes is steadily increasing worldwide [3, 4]. It is estimated that 700 million people will have diabetes by 2045 [5]. The number of patients with diabetic kidney disease is expected to increase alongside the prevalence of diabetes [5]. Patients with DN have a significant risk of cardiovascular disease and a competing risk for end-stage kidney disease [6, 7].The increasing number of DN patients and their cardiovascular complications urgently warrant a better understanding of the pathogenesis of DN to develop therapeutic agents that target specific mechanisms.

Hyperglycemia in the development of DN damages the kidney by producing advanced glycation products, hemodynamic disturbances, growth factors, and hormonal changes [8]. These multifactorial changes result in hyperglycemia-induced uncontrolled inflammatory and immune responses which are associated with acute and chronic forms of kidney damage [9, 10]. Diverse inflammatory cytokines, such as interleukin-1 (IL-1), IL-16, IL-18, and tumor necrosis factor-α (TNF-α), and chemokines have been identified to participate in the pathogenesis of DN [11, 12]. Impairment of signaling pathway (e.g. Janus kinase/signal transducer and activator of transcription, nuclear factor kappa-light-chain-enhancer of activated B cells, Nod-like receptor, Toll-like receptor pathway, etc.) is also involved in kidney inflammation and fibrosis, which is related to the development of DN and disease progression [13, 14]. Endothelial cells affected by hyperglycemia play a role in recruiting leukocytes as the expression of adhesion molecules is upregulated by inflammatory mediators [15]. Selectins, integrins, intercellular cell adhesion molecules-1 (ICAM-1) and vascular cell adhesion molecule-1 (VCAM-1) are involved in leukocyte transmigration into kidney interstitium and promote kidney damage [16]. Infiltrated leukocytes and accumulation of released cytokines in tissue accelerates the kidney injury by inducing oxidative stress and increasing vascular permeability [14, 17].

Neutrophils might play a multifaceted role in DN pathogenesis. Hyperglycemia, in the diabetic milieu, promotes neutrophil activation and prolongs its lifespan, thereby inhibiting their inflammatory responses [18–22]. In the development of DN, activated neutrophils persistently express integrin CD11b/CD18, allowing the interaction between neutrophil and endothelial cells [23–25]. These activated neutrophils exaggerate effector functions, such as reactive oxygen species (ROS) generation and proinflammatory cytokine production, thereby amplifying the inflammatory cascade within the renal parenchyma [26, 27]. A study in subjects with or without diabetic complication, superoxide production of neutrophil induced by TNF-α was significantly increased in patients with diabetic complications [28].

Neutrophil extracellular traps (NETs), composed of nuclear chromatin and granular contents, were first described as immune defense mechanism against pathogens [29]. Increasing evidence for NET-related pathology has been reported in diverse of disease entities including diabetes, autoimmune disease, and cancer [30–33]. Dysregulation of NETs can lead to disease pathology through several mechanisms such as direct cytotoxic effect, sterile inflammation, and vascular occlusion [34]. In diabetes, both in vitro and in vivo analysis revealed that high glucose induced NETs formation [22]. High glucose enhanced NETs formation, which contributes to the glomerular injury by inducing pyroptosis in glomerular endothelial cells in DN [35]. Moreover, these NETs directly induce podocyte and vascular injury [36], indicating the pivotal role of neutrophils in DN pathogenesis. However, the exact role of NETs in DN pathogenesis and disease progression is less understood considering conflicting data showing that IL-6 induced NETosis was suppressed by high glucose [37]. Thus, this study aimed to investigate the role of NETs in DN using diabetic murine models.

## Methods

### Animals

Mice were housed with free access to chow and water and were kept in SPF units with a 12-light/dark cycle. All animal experiments were approved by the Animal Care and Use Committee of Kyungpook National University (KNU-2023-0032). Type 2 Diabetic models used 10-week-old male BKS(D)-Lepr db/+JOrlRj témoin (db/m, *n* = 5) and BKS(D)-Lepr db/dbJOrlRj (db/db, *n* = 9) mice, which were purchased from Janvier Labs (Le Genest-Saint-Isle, France), given with either a regular diet (D10001, Research Diets Inc., New Brunswick, NJ, USA) of chow for 10 weeks starting at 10 weeks of age. At week 20, type 2 diabetic mice were anesthetized by 3–5% isoflurane. Type 1 Diabetic models used 5-week-old male C57BL/6 mice, which were obtained from Hyo-chang Science (Daegu, South Korea). Mice had fasted for 4 h before intraperitoneal streptozotocin (STZ, Sigma-Aldrich, St. Louis, MO, USA) administration at 50 mg/kg (in phosphate-buffered saline [PBS]) for 5 consecutive days (*n* = 9). The wild-type (WT) control mice were intraperitoneally administered PBS (*n* = 5). The animals were euthanized 8 and 12 weeks after STZ injection. Blood serum and kidney samples were harvested from all mice. The serum was subjected to blood urea nitrogen (BUN), creatinine (Cr), and glucose analysis. One kidney was quickly removed for histological and immunohistochemistry (IHC) staining, while the other was removed and stored at − 70℃ before the Western blot assay.

### Kidney function and histopathological studies

BUN, Cr, and glucose levels were evaluated in mouse serum by GCLabs (Yongin, Korea) using the Cobas 8000 modular analyzer system (Roche, Germany). Kidney tissues from each experimental group were immersion-fixed with 4% paraformaldehyde (pH: 7.4) and then embedded in paraffin. Three-micrometer tissue sections were prepared and stained with periodic acid-Schiff (PAS) using standard protocols to determine histological changes and glomerular size, respectively. Glomerular size (2 μm) was measured as the length of each glomerular using Image J (NIH) in > 9 randomly selected fields in the cortex sections and averaged.

### IHC analysis

Kidney tissues from each experimental group were immersion fixed with 4% paraformaldehyde (pH 7.4) and then embedded in paraffin blocks. The blocks were cut into 2-µm-thick sections. IHC staining detected neutrophil deposition and renal fibrosis in the kidney through primary antibodies incubated overnight at 4℃. The primary antibodies include the following: anti- myeloperoxidase (MPO) (1:50, ab9535, Abcam), anti-citH3 (1:200, ab5103, Abcam), anti- neutrophil elastase (NE) (1:100, ab68672, Abcam), anti-Ly6G (1:400, ab238132, Abcam), anti-Fibronectin (1:100, ab2413, Abcam), and anti-α-smooth muscle actin (SMA) (1:100, ab5964, Abcam). The section slides were incubated 1 h at room temperature with species-specific secondary antibodies and visualized by incubating with 3,3-diaminobenzidine (DAKO ChemMate Detection Kit) and hydrogen peroxide, and counterstained with Harris’ hema-toxylin. At least ten random sections from the cortex and outer medulla of each sample were quantified using Image J software (NIH), and the positive area was calculated and averaged. For the quantification of NETs in immunohistochemistry, citH3, a surrogate marker for NETs, was used along with MPO and NE, markers for neutrophil activation and recruitment as described previously [38]. Specimens were considered positive for NET when at least one tissue area showed moderate staining with the citH3 and at least one activation or recruitment marker, either MPO or NE. Additionally, the quantity was determined in a semi-quantitative fashion based on the extent of tissue staining compared to the negative control.

### Immunoblot analysis

Immunoblot analysis detected the marker proteins of fibrosis in the kidney. The tissues were lysed in RIPA buffer (50 mmol/L Tris-HCl [pH 8.0], 150 mmol/L NaCl, 1% NP-40, 0.5% sodium deoxycholate, and 0.1% sodium dodecyl sulfate) and Protease Inhibitor Cocktail Set III (Calbiochem, Darmstadt, Germany). Protein concentration was measured by Bradford’s method in lysate of tissues. We used 10% SDS-polyacrylamide gel electrophoresis to separate 20 mcg of protein and transferred it to a nitrocellulose membrane, which was blocked with 10% skimmed milk in 10 mmol/L Tris-buffered saline with 0.1% Tween 20 (TBS-T) for 1 h at room temperature followed by overnight incubation at 4℃ with diluted primary antibodies in TBS-T. The primary antibodies include the following: anti-fibronectin (1:5000, Ab2413, Abcam), anti-α-SMA (1:5000, Ab5694, Abcam), and anti-glyceraldehyde 3-phosphate dehydrogenase (1:2000, 2118 S, Cell Signaling). After washing, the membrane was incubated with a horseradish peroxidase-conjugated secondary antibody (Dako, Glostrup, Denmark) for 1 h at room temperature and detected using advanced ECL reagents (Amersham Bioscience, Piscataway, NJ, USA). An ImageQuant™ LAS 4000 system (GE Healthcare Life Sciences, Tokyo, Japan) was used to visualize the membranes. The Scion Image software (Scion, Frederick, MD, USA) was used to quantify the intensity of the bands.

### Cell cultures

We purchased Human Kidney-2 Cell Line (HK2) from the American Type Culture Collection (Virginia, USA). Conditionally immortalized glomerular mesangial cell line (CIHGM-1) and Conditionally immortalized glomerular endothelial cell line (ciGEnC) were purchased from Ximbio (London, UK). The cells were cultured in RPMI-1640 supplemented with 10% fetal bovine serum and antibiotic (10 U/ml of penicillin and 10 U/ml of streptomycin) at 37℃ in a humidified atmosphere of 5% CO2 and 95% air. The CIHGM-1 and ciGEnC cells were proliferated at a temperature of 33℃, and after transferred to a temperature of 37℃, the cells entered growth arrest and differentiated cells were used in the experiment.

### Quantification of NETs

Venous blood was collected from a healthy male donor into a heparinized vacutainer, and neutrophils were isolated using histopaque 1077, followed by dextran sedimentation and hypotonic lysis. For NETs isolation, neutrophils (1 × 10^8^) were stimulated with PMA (1 µg/ml) in a 100 mm cell culture dish at 37 °C for 1 h. After stimulation, the supernatant was removed, the cells were washed with ice-cold PBS, resuspended in RPMI 1640 (Gibco), and centrifuged at 2500 rpm for 30 min at room temperature. The supernatants containing NETs were collected and quantified according to the indicated cell numbers (1 × 10^8^ cells). For the viability assay, the supernatants containing NETs were diluted to maintain the relative ratio of neutrophil to kidney epithelial cell (5 × 10^6^ cells, E:T ratio = 20:1).

### Cell viability assay

The HK2, CIHGM-1, and ciGEnC cells were seeded in 96-well plates and incubated using a serum-free medium for 1 h and 24 h. We previously conducted a cytotoxicity test based on the E: T ratio dependence. The neutrophils were co-cultured with renal epithelial cells, an appropriate E: T ratio was selected that did not cause cytotoxicity. After starvation, the neutrophils were co-cultured at an E: T ratio of 20:1 (20:1), as well as the DNase-treated group (20:1 + DNase). Then, neutrophils were washed with 1X PBS three times to remove suspended neutrophils completely and only the renal cells were used for the assay [39–41]. The cell viability was analyzed using the Cell Counting Kit-8 (CCK-8) assay (Dojindo Laboratories, Kumamoto, Japan) following the manufacturer’s instructions. CCK-8 solution (10 µl) was added to each well of the culture medium. The plates were placed in a CO2 incubator for 1–4 h to react. The absorbance was measured at 450 nm using a microplate (SPARK 10 M, Tecan, Durham, NC, USA). The value was expressed as a percentage of control.

### Assessment of cell apoptosis

The FITC Annexin V Apoptosis Detection Kit (BD biosciences, USA) was used to detect apoptosis by flow cytometry. After treatments, the cells were trypsinized and gently centrifuged. The cell pellet was resuspended in 100 µL of 1x Annexin binding buffer and incubated with 5 µL of Annexin V and 5 µL of propidium iodide (PI) for 15 min in the dark. After adding 200 µl of PBS, the cells were analyzed on the FACScan (Becton Dickinson, Franklin Lakes, NJ, USA) in the Annexin–FITC and PI. The percentage of apoptotic cells was calculated as the ratio of Annexin V-positive and Annexin V/PI-double positive cells to total cells.

### Statistical analysis

Data represent mean ± standard error of the mean. The normality of the data was determined using the Shapiro–Wilk test. Comparison of parametric data was performed using one-way ANOVA with Tukey’s host hoc test. Non-parametric data was compared using the Kruskal–Wallis test with Mann–Whitney U-test, as a post-hoc test. Statistical significance was considered at *P* < 0.05. Statistical analysis was performed using GraphPad Prism 10.1.2 5.01 (GraphPad Software Inc., La Jolla, CA).

## Results

### Biochemistry analysis and renal pathological structures in DN model

**Fig. 1:**
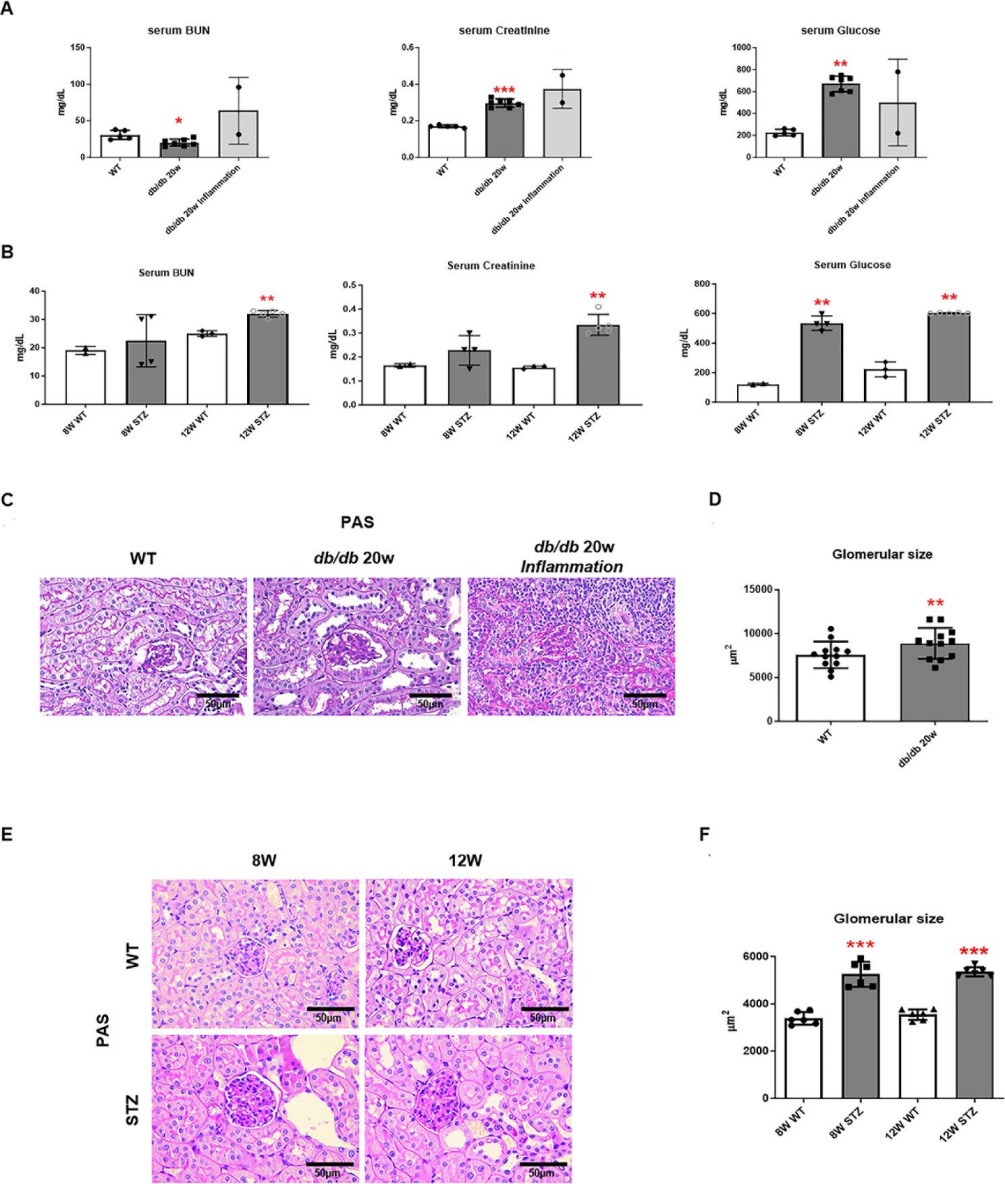
The biochemistry analysis of serum blood urea nitrogen (BUN), creatinine, and glucose levels and renal pathological structures in diabetic models. (A) Biochemistry analysis in db/db 20-week-old mice. *n* = 2–7. (B) Biochemistry analysis in streptozotocin (STZ)-induced diabetic models for 8-week- and 20-week-old mice. *n* = 5 each group. (C, E). Representative images of periodic acid-Schiff (PAS) staining (x400) and (D, F) glomerular size in both diabetic models. *n* ≥ 6. (Kruskal–Wallis test, *p < 0.05, **p < 0.01, ***p < 0.001 vs. WT)

We used two diabetic murine models. The type 1 diabetic model was established using STZ injection to C57B/L mice, and db/db mice were used for the type 2 diabetic model. The db/db mice were maintained for 20 weeks, with a significant increase in Cr levels, which is a kidney function measurement, along with an increase in serum glucose levels. Conversely, BUN levels were decreased compared to normal mice (WT) (Fig. 1A). Inflammatory db/db mice had increased BUN, Cr and serum glucose levels; however, statistical analysis could not be conducted due to an inadequate sample size (Fig. 1A). The STZ-induced diabetic model, which is a type 1 diabetic model, selected two serum glucose level elevation points to confirm kidney function at 8 and 12 weeks of STZ administration. Both the 8- and 12-week-old STZ-induced diabetic model groups demonstrated significantly increased BUN and Cr levels, thereby confirming the successful development of the hyperglycemic diabetic murine model (Fig. 1B). To observe the hyperglycemia-induced structural changes in the kidneys, the degree of kidney damage was quantified through PAS staining. The size of the glomeruli, which is the main unit of the kidney, was significantly increased in both types 1 and 2 DN models compared to the control group (Fig. 1C and E). Additionally, in the type 2 diabetes model, increased interstitial inflammation was observed in inflammatory db/db mice compared to non-inflammatory db/db mice (Fig. 1C). The glomerular size measured on the slides of each randomly selected group demonstrated a significant increase in size (Fig. 1D and F). These results indicated that hyperglycemia changes the glomerular size in the kidney and decreases the renal function in the types 1 and type 2 DN models.

### High glucose in serum levels induced the progression of fibrosis in diabetic kidney tissue

**Fig. 2:**
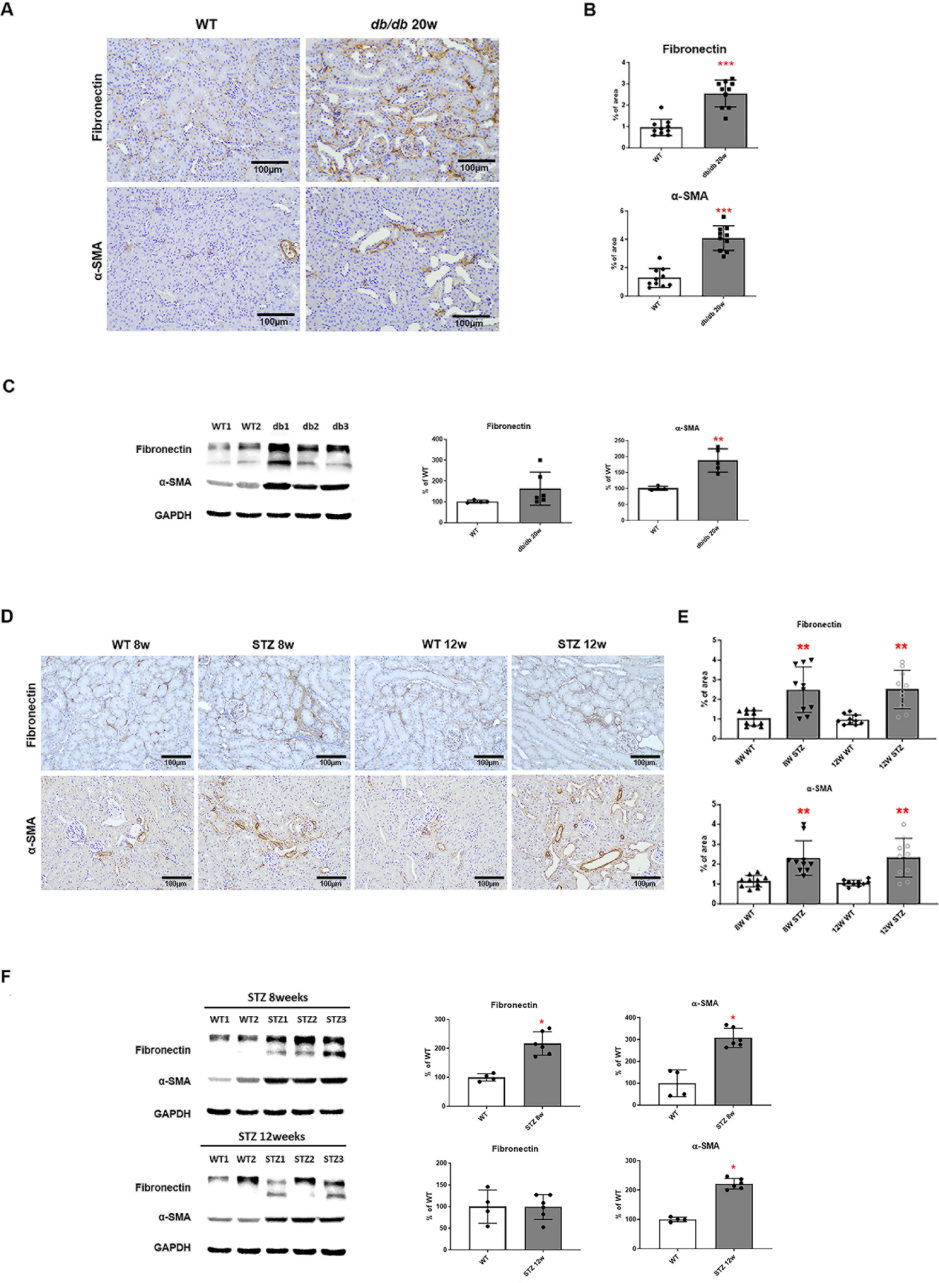
High glucose serum levels induced the progression of fibrosis in diabetic kidney tissue. (A, B) Representative images of fibronectin and α-SMA staining of kidney tissue in db/db 20-week-old mice (x200). Bar graph summarizing the % of the positive staining area. *n* = 10 each group. (C) Fibronectin and α-SMA protein levels in db/db 20-week-old mice were determined by Western blot. *n* = 4–6 each group. (D, E) Representative images of fibronectin and α-SMA staining of kidney tissue in STZ-induced diabetic models (x200). Bar graph summarizing the % of the positive staining area. *n* = 10 each group. (F) Fibronectin and α-SMA protein levels in STZ-induced diabetic models were determined by Western blot. *n* = 4–6 each group (The Kruskal–Wallis test, *p < 0.05, **p < 0.01, ***p < 0.001 vs. WT)

We examined the renal fibrosis and structural changes in the kidneys of diabetic mice. The kidneys of db/db mice groups were harvested and subjected to IHC staining analysis for fibrosis markers, fibronectin, and α-SMA. Compared to WT, kidney tissue from 20-week-old db/db mice showed increased positive cells for fibronectin and α-SMA (Fig. 2A and B). Quantitative IHC staining shows a significant increase in fibrosis and ɑ-SMA in diabetic mice groups compared to WT mice group. Examination of protein expression in kidney tissue revealed that α-SMA was also significantly increased in 20-week-old db/db mice (Fig. 2C).

Kidneys were harvested 8 and 12 weeks after STZ administration, and IHC staining analysis for fibrosis markers, fibronectin and α-SMA showed that diabetic mice were positive for fibronectin and α-SMA in the tubules. (Fig. 2D and E). Additionally, the expression of fibrosis markers was higher 8 weeks after STZ administration than after 12 weeks. At the same time, protein levels of fibronectin and α-SMA increased (Fig. 2F). These results indicate that hyperglycemia accelerates renal fibrosis along with structural changes in the kidney.

### Expression patterns of NET-related proteins, MPO, citH3, NE, and Ly6G in DN models

**Fig. 3:**
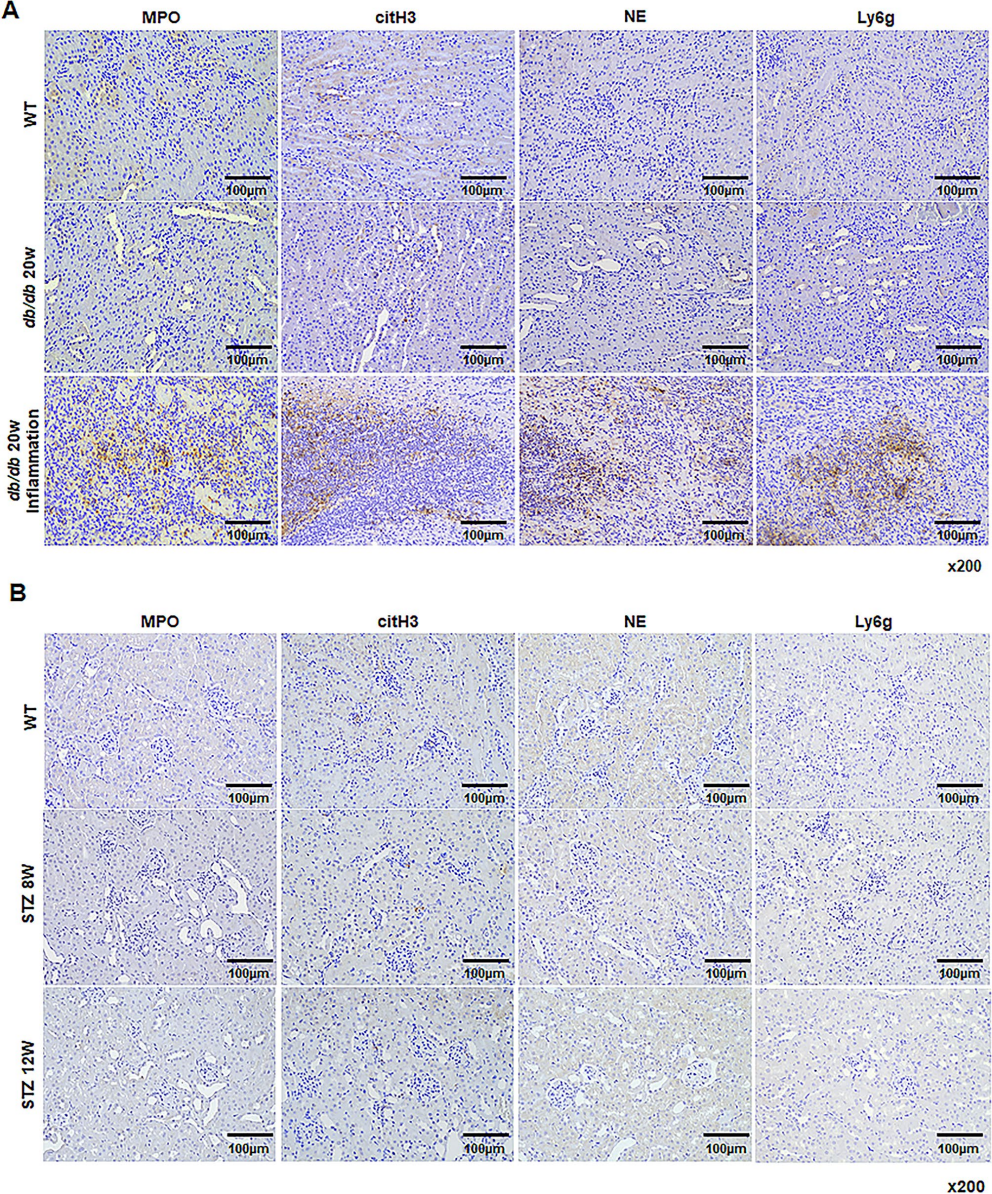
The expression patterns of NETs-related proteins in a model with DN or inflammatory DN. (A) Representative images of NETs-related proteins staining of kidney tissues in db/db mice. (B) Representative images of NETs-related proteins staining of kidney tissues in STZ-induced diabetic models (x200)

We further examined neutrophil deposition and NET formation in the kidney under diabetic conditions. Neutrophils were identified using Ly6G, while NET formation was assessed using the NET-related proteins, including MPO, citH3, and NE. We observe neither neutrophils nor NETs in the kidneys of WT mice and 20-week-old db/db mice. However, all NET-related proteins were significantly increased in the kidneys of diabetic mice, which were inflamed due to long-term maintenance of hyperglycemia (Fig. 3A). We found no NET deposition in the kidney in the STZ-induced diabetic model despite decreased renal function and fibrosis (Fig. 3B). These results indicate that NET formation is triggered by inflammation resulting from high serum glucose.

### Apoptosis and viability with NETs or NET blockade in renal cells

**Fig. 4:**
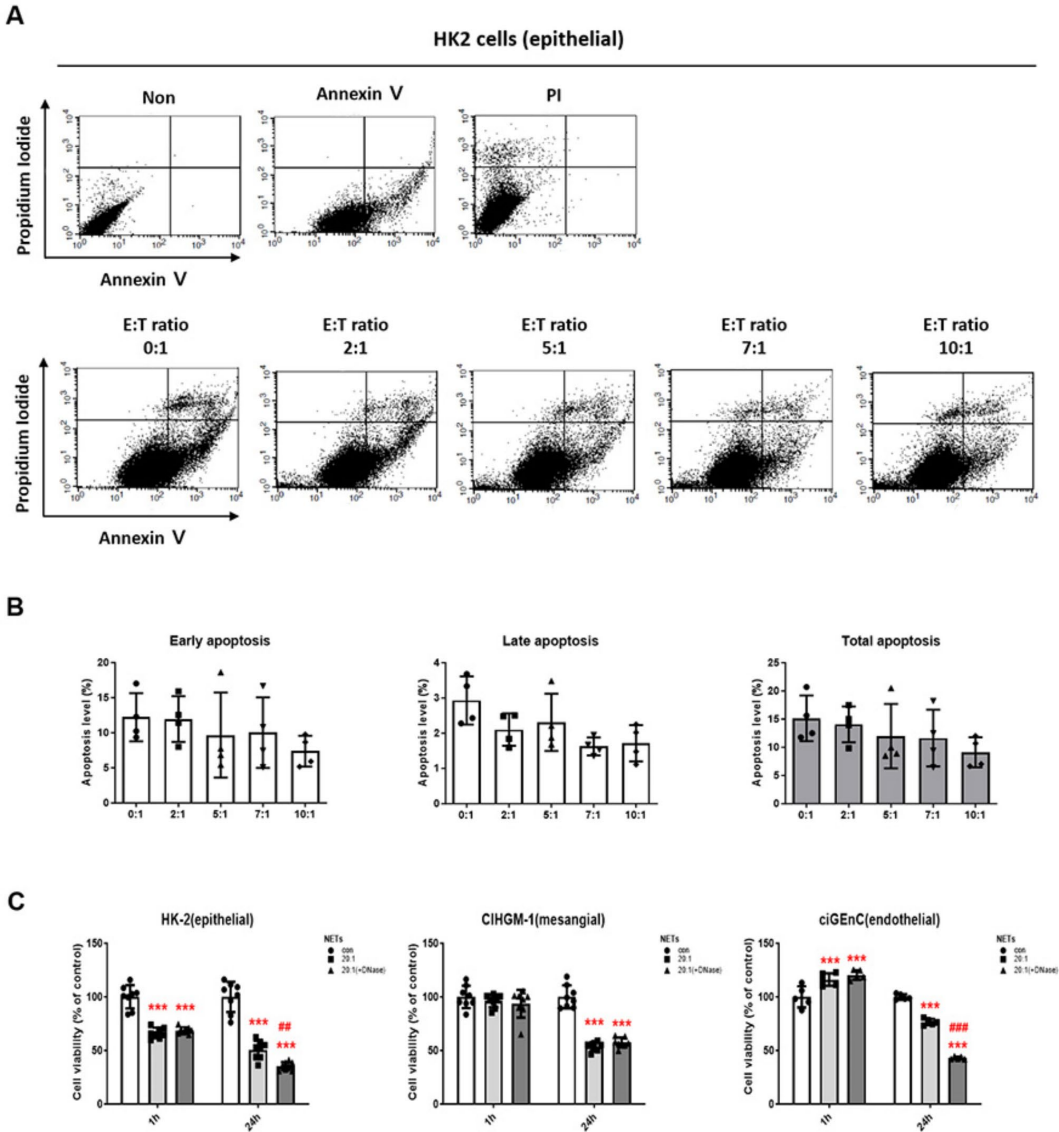
Apoptosis and viability with NET or NET blocking in HK2 cells. (A) Annexin V-FITC and propidium iodide (PI) staining and (B) quantitative analyses for apoptosis after co-culture of neutrophils in HK2 cells. *n* = 4 each group. (C) MTT assay results according to NET blocking in HK2, CIHGM-1, and ciGEnC cells. *n* = 8 each group. (One-way ANOVA, ***p < 0.001 vs. con, ## *p* < 0.01, *p* < 0.001 vs. 20:1)

We then investigated the cytotoxic effects of neutrophils on various kidney cells. Neutrophils were co-cultured with HK2 cells at various effector-to-target (E: T) ratios and the apoptotic rates of HK2 cells were measured using annexin V and PI staining to examine the optimal E: T ratio. HK2 cells demonstrated no significant changes in the apoptotic rates up to an E: T ratio of 10:1 (Fig. 4A and B), thus next treatments to kidney cells were co-cultured with NET collected from neutrophils at an E: T ratio of 20:1. We investigated cell viability in response to NET effects in human kidney cells HK2, stromal cells CIHGM-1, and endothelial cells ciGEnC. Each cell was co-cultured with NET collected from neutrophils at 20:1 or DNase-treated inactivated NETs collected from neutrophils were co-cultured at 20:1. The cell viability of HK2 cells decreased after 1 h of culture, and the survival rate was significantly decreased in both NET co-culture and inactive NET co-culture at both 1 h and 24 h (Fig. 4C, left panel). The CIHGM-1 cells showed a decrease in survival rate when cultured with NET for 24 h; however, co-cultured with inactive NET at 24 h did not enhance cell viability (Fig. 4C, middle panel), suggesting that NETs may not induce the decrease in cell viability. In ciGEnC cells, DNase treatment did not affect the cytotoxic function of NET, showing a significant decrease at 24 h of culture (Fig. 4C, right panel). This indicates the minimal role of NETs on cellular damage in the kidney.

## Discussion

In the present study, we induced DN using type 1 and 2 murine models. We revealed a significant increase in NET-related proteins in diabetic mice with interstitial inflammation induced by prolonged hyperglycemia. In contrast, NETs were absent in the non-inflammatory diabetic mice. The treatment with NETs reduced the viability of kidney tubular cells, but blocking NETs did not attenuate cell cytotoxicity. The exclusive presence of NETs in mice with tubulointerstitial inflammation indicates a direct association between NETs and the inflammatory response during the development of DN.

The role of NETs in organ injury, such as acute kidney injury [42, 43] has been reported along with their potential contributions to autoimmune kidney disease, including lupus nephritis [44, 45] and anti-neutrophil cytoplasmic autoantibody-associated vasculitis [46, 47], have been described. This accumulating evidence emphasizes the emerging role of NETs in sterile inflammation. Indeed, neutrophils are primed for NET formation in patients with diabetes [20] and the concentration of dsDNA correlates the presence of DN [22]. Traditionally, the development of DN attributed to hemodynamic and metabolic disorders, including oxidative stress and advanced glycation end-product [48].

However, some evidence suggests the involvement of immune and inflammatory responses from neutrophils in the pathogenesis of DN. Recent studies have revealed significantly increased NETs formation in glomeruli of diabetic mouse models and humans [35, 49]. High glucose levels induce the formation of NETs, leading to endothelial dysfunction through inflammasome activation. Furthermore, NET formation amplifies the level of cleaved IL-1β and promotes NLR family pyrin domain containing 3 inflammasome activation [49]. Inhibition of peptidylarginine deiminase 4 (PAD4), a key enzyme involved in NETs formation, has been shown to reduce NETs formation and associated albuminuria [49]. Another study demonstrated that hyperglycemia-induced NETs exerted cytotoxic effects, thereby promoting pyroptosis in endothelial cells, and the knockout of PAD4 ameliorated endothelial injury and albuminuria [35]. Furthermore, transcriptome analysis and electron microscopy demonstrated that NET induces pyrolysis in glomerular endothelial cells, which was supported by increased markers of pyrolysis in endothelial cells from diabetic mice and DKD patients [35]. In a recent study analyzing NET-related genes (NRGs), increased expression of CASP1 and LYZ were significantly associated with tubulointerstitial injury in DN [50]. These findings, which reveal a link between NRGs and tubulointerstitial injury in DN, are aligned with our results and underscore the need for further mechanistic research.

Our study revealed that the diabetic mouse models demonstrated elevated serum Cr levels and signs of hyperglycemia-induced glomerular lesions, such as glomerular hypertrophy and tubulointerstitial fibrosis. These results are consistent with the early phase of DN, as the changes in kidney structure were predominantly limited to glomeruli, thereby preserving most of their integrin [51]. Additionally, the decreased or similar BUN levels in db/db mice and 8-week-old STZ-induced diabetic model, compared to control groups supported the results. Although our study successfully developed DN animal models, NETs were not consistently observed in these animal models, differing from previous studies [15, 16]. These results suggest that the role of neutrophils, specifically through the formation of NETs, in DN is complex and dependent on inflammation. While the traditional understanding of DN pathogenesis focuses on hemodynamic and metabolic factors, recent insights highlight the significance of inflammatory and immune responses, particularly those mediated by neutrophils. Our findings, which show elevated NET-related proteins in diabetic mice with tubulointerstitial inflammation but not non-inflammatory diabetic mice, suggest that NET formation from neutrophils depends on inflammatory status rather metabolic status in DN pathogenesis. Moreover, the absence of NETs in early-stage diabetic mice models indicates to their limited role in the initial phases of DN and suggests that NETs might be associated with later stages or disease progression, rather than the early onset of DN. Furthermore, our observation that blocking NETs did not attenuate cell cytotoxicity highlights the complexity of the inflammatory mechanisms in DN pathogenesis. While NETs are an important component of the inflammatory response in DN, they might not be sole mediators of tissue damage. This complexity requires further research to dissect the precise roles of neutrophils and NETs at various stages of DN and their interactions with other pathophysiological factors. Therefore, we can speculate that neutrophils and NETs are significant players in the inflammatory landscape of DN. Their involvement is more pronounced in the inflammatory response and might be prominent in the progressive stages of disease, suggesting potential targets for therapeutic intervention.

Our study has several limitations. First, we were unable to measure the urine albumin-to-Cr ratio in our animal models. Albuminuria, which is known to correlate positively with MPO-DNA complexes [35], is used to assess the severity of DN in relation with NETs formation. However, our mouse models demonstrated glomerular hypertrophy and a mild degree of tubulointerstitial fibrosis, which are both characteristics of early DN stages. Secondly, NETs were observed only in mice with hyperglycemia-induced inflammatory DN. Further characterization of hyperglycemia-induced inflammation through additional inflammatory markers or pathological analyses would have been helpful in understanding the role of NETs in DN. The varied pathological results in our mice model under identical conditions could be associated with different susceptibilities to DN. Further pathophysiological characterization of these animal models could enhance our understanding of the role of NETs in DN development and/or progression. Lastly, NETs were observed in the kidney interstitium using IHC staining. Consequently, these NETs caused by an inflammatory response may be associated with CKD progression, rather than being a major DN pathogenesis. It is important to determine whether glomerular endothelial cells are affected by hyperglycemia for the development of diabetic mouse model for NET formation.

## Conclusions

We investigated the pathological role of NETs in the development of DN. NET-related proteins were only expressed in the DN model with tubulointerstitial inflammation. NETs inhibition did not attenuate significant changes in kidney cell apoptosis. These findings emphasized the role of NETs formation in kidney injury with inflammation.

## Electronic supplementary material

Below is the link to the electronic supplementary material.

Supplementary Material 1

## Data Availability

The datasets used and/or analysed during the current study are available from the corresponding author on reasonable request.

## Abbreviations

BUN: Blood urea nitrogen
CCK-8: Cell Counting Kit-8
ciGEnC: Conditionally immortalized glomerular endothelial cell line
CIHGM-1: Conditionally immortalized glomerular mesangial cell line
citH3: Citrullinated histone H3
CKD: Chronic kidney disease
Cr: Creatinine
DN: Diabetic nephropathy
HK2: Human Kidney-2 Cell Line
ICAM-1: Intercellular cell adhesion molecules-1
IHC: Immunohistochemistry
IL-1: Interleukin-1
Ly6G: Lymphocyte antigen 6 complex locus G6D
MPO: Myeloperoxidase
NE: Neutrophil elastase
NETs: Neutrophil extracellular traps
PAD4: Peptidylarginine deiminase 4
PAS: Periodic acid-Schiff
PBS: Phosphate-buffered saline
PI: Propidium iodide
ROS: Reactive oxygen species
SMA: Smooth muscle actin
STZ: Streptozotocin
TNF-α: Tumor necrosis factor-α
TUNEL: Terminal deoxynucleotidyl transferase-mediated dUTP nick end labeling
VCAM-1: Vascular cell adhesion molecule-1
WT: Wild-type
