# Supplementary material for: Observation of neutrophil extracellular traps in the development of diabetic nephropathy using diabetic murine models
Source: Lab Anim Res. 2024 Nov 7;40:38. doi: 10.1186/s42826-024-00226-2 (PMC11542270; doi:10.1186/s42826-024-00226-2)
Supplement: Supplementary file 1 — Supplementary Material 1 [file 42826_2024_226_MOESM1_ESM.pdf]

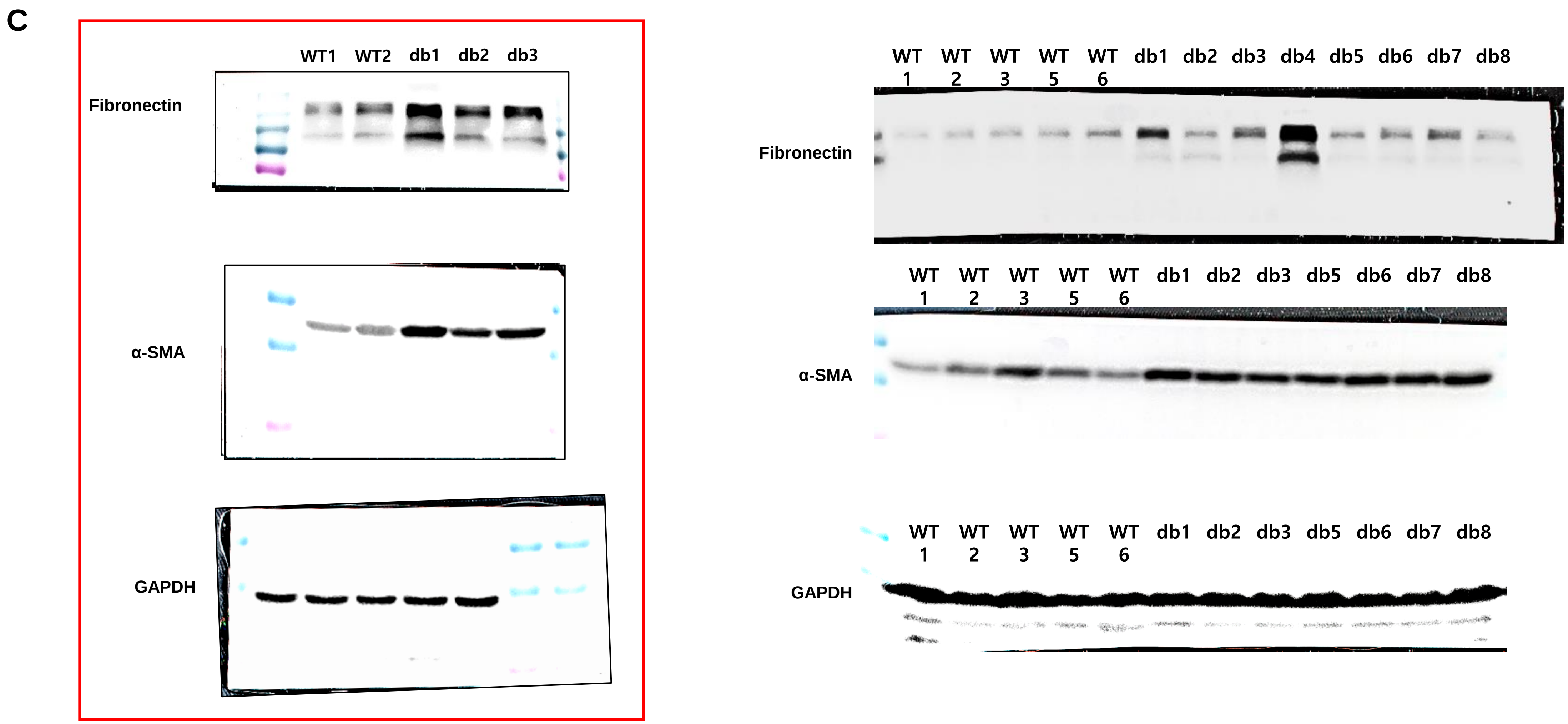

Figure 2. High glucose serum levels induced the progression of fibrosis in diabetic kidney tissue. (C) Fibronectin and α-SMA protein levels in db/db 20-week-old mice were determined by Western blot. n = 4–6 each group. (F) Fibronectin and α-SMA protein levels in STZ-induced diabetic models were determined by Western blot. n = 4-6 each group.

F

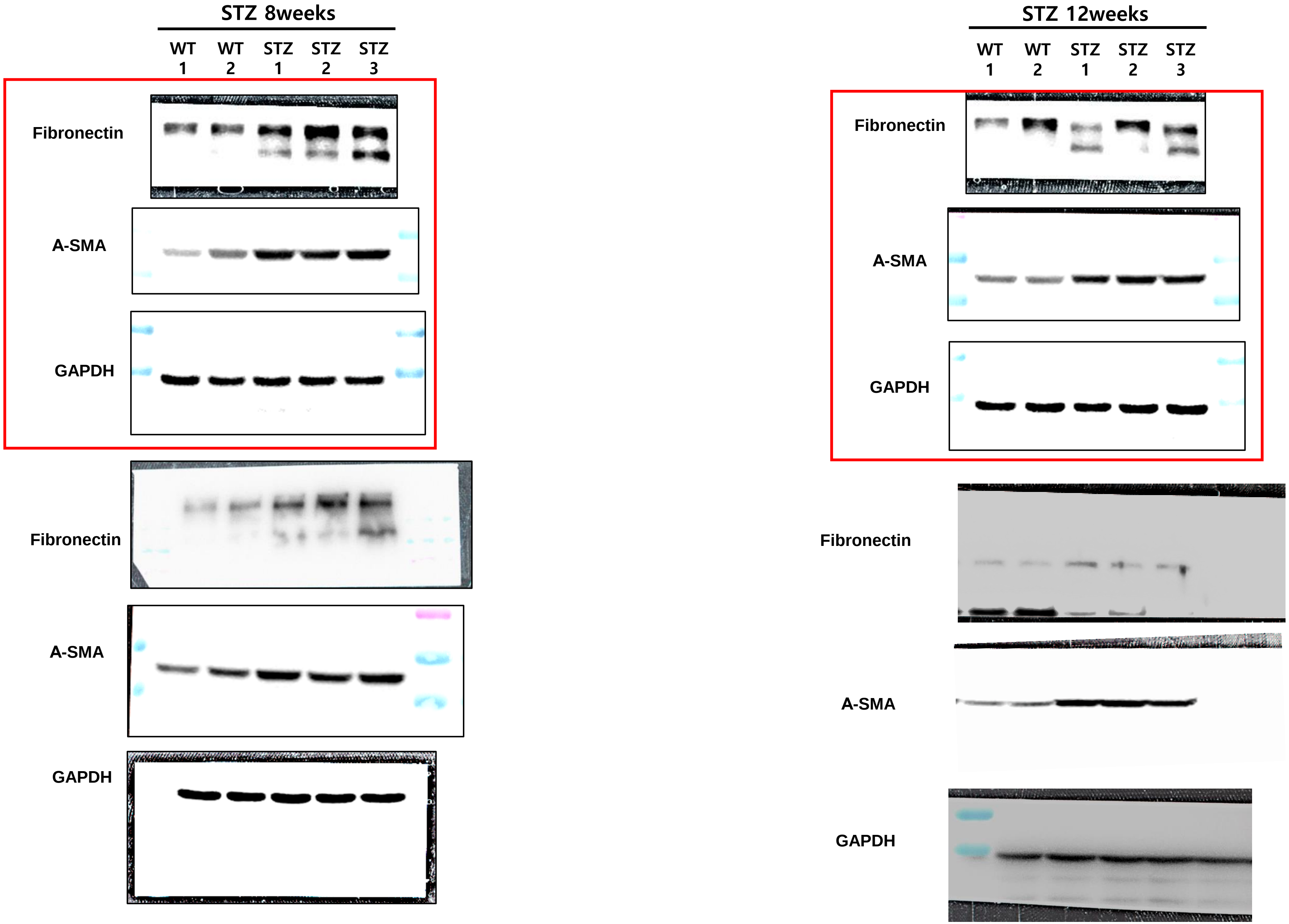
STZ 8weeks
WT 1
WT 2
STZ 1
STZ 2
STZ 3
Fibronectin
A-SMA
GAPDH
Fibronectin
A-SMA
GAPDH
STZ 12weeks
WT 1
WT 2
STZ 1
STZ 2
STZ 3
Fibronectin
A-SMA
GAPDH
Fibronectin
A-SMA
GAPDH
